# Transmigration of polymorphnuclear neutrophils and monocytes through the human blood-cerebrospinal fluid barrier after bacterial infection *in vitro*

**DOI:** 10.1186/1742-2094-10-31

**Published:** 2013-02-28

**Authors:** Ulrike Steinmann, Julia Borkowski, Hartwig Wolburg, Birgit Schröppel, Peter Findeisen, Christel Weiss, Hiroshi Ishikawa, Christian Schwerk, Horst Schroten, Tobias Tenenbaum

**Affiliations:** 1 Department of Pediatrics, Pediatric Infectious Diseases, Medical Faculty Mannheim, Heidelberg University, Theodor-Kutzer-Ufer 1-3, Mannheim, 68167, Germany; 2 Institute of Pathology and Neuropathology, University of Tuebingen, Tuebingen, Germany; 3 Natural and Medical Sciences Institute Reutlingen, Reutlingen, Germany; 4 Institute for Clinical Chemistry, Medical Faculty of Mannheim, Heidelberg University, Mannheim, Germany; 5 Department of Statistics, Medical Faculty Mannheim, Heidelberg University, Mannheim, Germany; 6 Department of NDU Life Sciences, School of Life Dentistry at Tokyo, The Nippon Dental University, Chiyoda-ku, Tokyo, Japan

**Keywords:** Blood-cerebrospinal fluid barrier, Leukocyte, Transmigration, Meningitis

## Abstract

**Background:**

Bacterial invasion through the blood-cerebrospinal fluid barrier (BCSFB) during bacterial meningitis causes secretion of proinflammatory cytokines/chemokines followed by the recruitment of leukocytes into the CNS. In this study, we analyzed the cellular and molecular mechanisms of polymorphonuclear neutrophil (PMN) and monocyte transepithelial transmigration (TM) across the BCSFB after bacterial infection.

**Methods:**

Using an inverted transwell filter system of human choroid plexus papilloma cells (HIBCPP), we studied leukocyte TM rates, the migration route by immunofluorescence, transmission electron microscopy and focused ion beam/scanning electron microscopy, the secretion of cytokines/chemokines by cytokine bead array and posttranslational modification of the signal regulatory protein (SIRP) α via western blot.

**Results:**

PMNs showed a significantly increased TM across HIBCPP after infection with wild-type *Neisseria meningitidis* (MC58). In contrast, a significantly decreased monocyte transmigration rate after bacterial infection of HIBCPP could be observed. Interestingly, in co-culture experiments with PMNs and monocytes, TM of monocytes was significantly enhanced. Analysis of paracellular permeability and transepithelial electrical resistance confirmed an intact barrier function during leukocyte TM. With the help of the different imaging techniques we could provide evidence for para- as well as for transcellular migrating leukocytes. Further analysis of secreted cytokines/chemokines showed a distinct pattern after stimulation and transmigration of PMNs and monocytes. Moreover, the transmembrane glycoprotein SIRPα was deglycosylated in monocytes, but not in PMNs, after bacterial infection.

**Conclusions:**

Our findings demonstrate that PMNs and monoctyes differentially migrate in a human BCSFB model after bacterial infection. Cytokines and chemokines as well as transmembrane proteins such as SIRPα may be involved in this process.

## Introduction

Bacterial meningitis still displays a life-threatening disease causing mortality and morbidity worldwide [1]. In this context, the role of the blood-cerebrospinal fluid (CSF) barrier (BCSFB) and the blood–brain barrier (BBB) is under investigation [2,3]. In bacterial meningitis, bacteria have to cross either the endothelium of the BBB or the epithelium of the BCSFB [1]. The epithelial cells of the choroid plexus (CP) form the main barrier of the BCSFB and are strongly connected through tight junctions (TJs) [4,5]. These TJs are formed by transmembrane proteins like occludin, claudins and zonula occludens (ZO)-1, that connect the TJ proteins with the actin cytoskeleton [4,5]. Still, pathogens may overcome those under particular circumstances.

*Neisseria meningitidis* (*N*. *meningitidis*) is a fastidious, aerobic gram-negative, capsule-expressing invasive pathogen. The different structures of the polysaccharide capsule and the immunogenicity designate the 13 different serogroups [6]. *N*. *meningitidis* frequently colonizes the nasopharynx. However, in a small percentage of patients, bacteria gain entry into the bloodstream and penetrate into the CNS via the BCSFB and the BBB to cause meningitis. The presence of *N*. *meningitidis* in vessels close to the choroid plexus suggests that bacteria may reach the CSF though the CP epithelium, but direct evidence for this is still lacking [7,8]. The pathogen employs different virulence factors such as the capsule, which enables the pathogen to survive within the bloodstream and reach the BCSFB and BBB. However, *N*. *meningitidis* can be either encapsulated or not, but only encapsulated invasive strains have ever been found in blood or the CSF [9]. Previous work from our group demonstrated a role of the *N*. *meningitidis* capsule to promote invasion into a human *in vitro* model of the BCSFB [10].

In the course of bacterial CNS infection different proinflammatory proteins such as TNFα attract leukocytes to the site of infection [11]. Previous studies showed an increased transmigration rate of polymorphonuclear neutrophils (PMNs) into the subcellular spaces as the first line of defense, promoted by an IL-8 release of epithelial or endothelial cells [12,13]. In this step two possible routes of leukocyte transmigration exist: the paracellular route [12,14], where the leukocytes overcome the TJs migrating between the cells involving a zipper-like mechanism [13] and the transcellular migration route, where the leukocyte migrates through the barrier-forming cell itself [15]. Previously we demonstrated that PMNs preferentially transmigrate via the transcellular route through primary porcine choroid plexus epithelial cells (PCPEC) [16].

The mechanism of transmigration depends on leukocyte type, species and host cell factors such as integrins, for example, ICAM1 [11,13]. During PMN and monocyte migration surface molecules, such as, CD11b/CD18 and CD47, and also transmembrane proteins, such as, signal regulatory protein (SIRP)α have been shown to be involved [17]. SIRPα, also known as SHPS-1, SIRPA, p84 and BIT, is selectively expressed on innate myeloid cells that include neutrophils, mast cells, dendritic cells, macrophages and monocytes [18-20]. The extracellular domain of SIRPα consists of three immunoglobulin (Ig) domains with 15 N-linked glycosylation sites [21]. Further differences in molecular mass have been observed between myeloid SIRPα (110 kDa) and neuronal SIRPα (85 to 90 kDa), which suggests a tissue-specific glycosylated form of SIRPα. The different glycosylation levels were shown to play a role in the binding capacity of SIRPα to CD47 [22]. The CD47 protein represents the extracellular ligand of SIRPα. It is known that the interaction of CD47/SIRP-α positively controls DC and innate cell migration across the endothelium [23]. These transmembrane glycoproteins and cytokines, as well as chemokines, may orchestrate an increased influx of leukocytes during and after *N*. *meningitidis* infection in humans.

Until recently no human *in vitro* model of the BCSFB existed to investigate transmigration of leukocytes over the BCSFB. The previous establishment of an inverted transwell filter system with human malignant choroid plexus papilloma cells (HIBCPP) that have high barrier characteristics [10,24], enables basolateral infection of host cells as well as investigation of leukocyte transmigration (TM) of leukocytes from the pathophysiologically relevant blood side to the apical CSF side. In the present study, we investigated the influence of *N*. *meningitidis* infection of HIBCPP onto the TM of freshly isolated PMNs and monocytes.

## Methods

### Cell culture

The HIBCPP was maintained as previously described [10,24]. In brief, HIBCPP were cultured in DMEM/HAM’s F12 nutrient mixture at a ratio of 1:1 (Invitrogen, Carlsbad, Germany) supplemented with 4 mM L-glutamin, 5 μg/ml insulin, 100 U/ml penicillin and 100 μg/ml streptomycin, and additional 15% of heat-inactivated FCS. Cells were seeded on transwell filter inserts (pore diameter 3.0 μm, pore density 2.0 × 10^6^ pores per cm^2^, growth area 0.33 cm^2^) (Greiner Bio-one, Frickenhausen, Germany) with a density of 0.7 × 10^5^. Hereafter, cells were cultured for two days before turning, and were cultured for up to 5 to 7 additional days before they were used for an experiment.

### Determination of barrier function

The measurement of the transepithelial electrical resistance (TEER) and the paracellular permeability provide information about the barrier characteristics. To analyze the barrier function throughout the experiments the TEER was documented by measuring the HIBCPP with a volt-ohm meter using the STX-2 electrode system (Millipore, Schwalbach, Germany) before the infection with bacteria, as well as before and after the TM of leukocytes [16]. Paracellular permeability was determined with dextran-TexasRed (MW 3000, Sigma, Deisenhofen, Germany). Dextran was added to the upper compartment of the filter together with the leukocytes for the 4-h incubation period in a concentration of 100 μg/ml, and flux across the HIBCPP monolayer in the basolateral-to-apical direction was measured. The concentration of dextran in the lower compartment was analyzed with a Tecan Infinite M200 Multiwell reader (Tecan, Männedorf, Switzerland). All measurements were performed in duplicate [16].

### Bacterial strains

The *N*. *meningitidis* strain, MC58 WUE 2135 (pil + opc + cap+) [25], and the unencapsulated mutant, MC58ΔsiaD (WUE2425; pil + opc + cap-) [26], as well as the constitutively unencapsulated strain α14 (pil + opc + cap-) [27,28] were kindly provided by U Vogel and H Claus (Institute for Hygiene and Microbiology, University of Würzburg, Germany), and were cultured as previously described. In brief, aliquots of bacteria were taken from −80°C, and plated on chocolate agar with vitox (Oxoid, Wesel, Germany) and grown at 37°C in 5% CO_2_ atmosphere overnight, followed by an additional 90-minute growth period in proteose peptone medium supplemented with 0.042% NaHCO_3_, 0.01 M MgCl_2_ and 1% PolyViteX (bioMerieux, Lyon, France) until the mid-logarithmic phase was reached and diluted to an optical density (OD)_600_ of approximately 0.1.

### Infection assays

Prior to the infection experiments HIBCPP were washed and transferred to medium without antibiotics supplemented with 1% FCS. At this time the cells had a TEER of about 55 Ω × cm^2^ ± 25 Ω × cm^2^. The change of serum concentration leads to a TEER increase up to 200 to 300 Ω × cm^2^ on the following day, and thereafter, cells were used for the TM experiment. HIBCPP on transwell filters were infected from the basolateral cell side (apical filter compartment) with MC58, MC58ΔsiaD or strain α14 at a multiplicity of infection (MOI) of 10. The infection was performed for 2 h at 37°C in 5% CO_2_ atmosphere. After the incubation time bacteria were killed by the addition of 100 U/ml penicillin and 100 μg/ml streptomycin to the transwell filters and further incubated for 22 h.

### Isolation of PMNs and monocytes

For immune cell transmigration, PMNs and monocytes were isolated from ethylenediaminetetraacetic acid (EDTA)-anticoagulated peripheral blood of healthy adult donors (approval was provided by the local ethics committee of the Medical Faculty of Mannheim, Heidelberg University (2009-327 N-MA)). PMNs were thereafter purified using Polymorphprep™ density sedimentation according to the manufacturer’s instructions (Axis Shield, Liverpool, United Kingdom). The interface containing PMNs was washed with Hank's Balanced Salt Solution (HBSS) with CaCl_2_ and MgCl_2_ (Invitrogen) followed by an erythrocyte lysis for 15 minutes (lysis buffer contains 8.3% of NH_4_Cl (Sigma-Aldrich Chemie GmbH, Steinheim, Germany), 1% KHCO_3_ (Sigma-Aldrich), 1% (w/v) EDTA (Biochrom AG, Berlin, Germany) dissolved in H_2_O and adjusted to 1 l and a pH of 7.2 to 7.4). For TM assays PMNs were loaded with fluorochrome 2',7'-bis-(2-carboxyethyl)-5-(and-6)-carboxyfluorescein, acetomethyl ester (BCECF-AM; Molecular Probes, Eugene, OR, USA) according to the manufacturer's instructions. The purity of isolated PMNs CD66b was 93.99% ± 1.91%.

For monocyte experiments PBMCs were isolated by Ficoll-Hypaque density gradient centrifugation. The negative isolation of monocytes was performed using the Dynabeads® Untouched™ Human Monocytes kit (Invitrogen), according to the manufacturer’s instruction, resulting in a purity of 91.53% ± 1.78% CD14^+^ cells. Monocytes were labeled in a similar fashion as described for PMNs.

### Transmigration assay

To analyze the TM process over HIBCPP, BCECF-AM-loaded leukocytes were added to the upper compartment of transwell filters (blood-side) in a ratio of PMNs:HIBCPP of 10:1 and monocytes:HIBCPP of 1:1, 22 h after previous stimulation of HIBCPP cells with bacteria, TNFα 10 ng/ml, or unstimulated control cells. As a chemoattractant for PMNs, IL-8 (10 ng/ml) (R&D, Wiesbaden, Germany) and for monocytes MCP-1 (50 ng/ml) (R&D) were used and applied in the lower compartment of the transwell filter 30 minutes before starting the TM experiments. After 4 h of TM the HIBCPP transwell filters were transferred for further analysis into a fresh plate, and the fluid within the lower filter compartment was centrifuged (5 minutes, 300 × g) to assure the attachment of PMNs or monocytes to the bottom of the wells. The centrifuged leukocytes were washed twice with HBSS with CaCl_2_ and MgCl_2_ (5 minutes, 300 × g), lysed with 1% Triton X-100 in PBS and quantified by fluorescence measurement with a Tecan 200 M Infinite Multiwell reader [16]. The remaining supernatant was used in some experiments for cytokine and chemokine, or dextran analysis.

### Immunocytochemistry

The transwell filters were stained as described previously [16]. In brief, transwell filters were cut out of the insert at the end of the experiment, washed with 1% HIBCPP medium and fixed with 4% formaldehyde for 10 minutes. The filters were permeabilized for 1 h with 0.5% Triton X-100/1% BSA in PBS and incubated with the primary antibodies at 4°C overnight to stain the TJ protein ZO-1 (dilution 1:250) (Invitrogen). Subsequently, filters were stained for 1 h with the secondary antibody (Alexa fluor® 594 goat anti-chicken, dilution 1:250) (Invitrogen), with phalloidin Alexa fluor® 660, dilution 1:60 (Invitrogen) for the actin cytoskeleton staining, with 4'-6-diamidino-2-phenylindole dihydrochloride (DAPI), dilution 1:50000 (Calbiochem, Darmstadt, Germany) and immune cell marker CD14, dilution 1:200 (BD Bioscience, Heidelberg, Germany) for monocytes or CD66b (Lifespan Biosciences, Seattle, WA, USA) for PMNs. After washing with PBS the filters were embedded in ProLong Antifade Reagent (Invitrogen). Subsequently, immunofluoresence analyses were performed using a Zeiss Apotome® with a 63×/1.4 NA objective lens and the Zeiss Scanning Software 4.6 Axiovision software Inside 4D (Carl Zeiss, Jena, Germany).

### Transmission electron microscopy

For the transmission electron microscopic analysis of HIBCPP and leukocytes, filters were fixed after TM for at least 4 h in a 2% glutaraldehyde solution in 75 mM cacodylate buffer (pH 7.4) and washed twice after the incubation time with 75 mM cacodylate buffer (pH 7.4). Subsequently, the support films were removed from the wells using a sharp ophthalmic scalpel. The filters were then cut into strips and post fixed in 1% osmium tetroxide (OsO_4_) in cacodylate buffer for 1 h, and dehydrated in ascending series of ethanol and propyleneoxide. For contrast enhancement, they were bloc-stained in uranyl-acetate in 70% ethanol for 4 h and flat-embedded in Araldite (Serva, Heidelberg, Germany). Using an ultramicrotome (Ultracut R, Leica, Bensheim, Germany), semi- (1 μm) and ultra-thin (50 nm) sections were cut. Ultra-thin sections were stained with lead citrate, mounted on copper grids and finally analyzed with a Zeiss EM 10 (Oberkochen, Germany) electron microscope.

### Focused ion beam/scanning electron microscopy (FIB/SEM)

For FIB/SEM analysis, the block of the embedded sample was sputter coated with gold palladium and mounted on an appropriate SEM (scanning electron microscope) sample holder. A semi-thin section of the embedded sample was imaged with the light microscope and correlated with the SEM image of the ultramicrotome block face to define the region of interest for three-dimensional analysis. Using a Crossbeam instrument (Zeiss) equipped with a gallium FIB (focused ion beam) and a low voltage SEM, FIB/SEM serial sectioning tomography [29,30] was accomplished. Thereby, the gallium FIB produces a series of cross-sections containing the region of interest. Each of these cross-sections is imaged by the low keV SEM using the energy-selected backscattered (EsB) electron detector for image acquisition, which yields to images with exceptionally high contrast due to the staining. Additionally, the images show no FIB-induced artifacts, such as curtaining, because of the missing topographical information in backscattered electron images. Using typical voxel sizes in the range of 10 × 10 × 10 nm, morphological information with high spatial resolution is obtained in the volume of interest.

The resulting stack of two-dimensional images was utilized for three-dimensional reconstruction using appropriate software. The open source software ImageJ [31], equipped with the plug-ins StackReg [32] VolumeJ [33] and 3D Viewer [34] was chosen for image processing and three-dimensional reconstruction of the transwell cell culture.

### Western blot

For western blot analysis protein lysates were generated at the end of the TM experiments by extraction of the leukocytes from the upper and lower compartment with modified radioimmunoprecipitation assay (RIPA) buffer (containing 50 mM Tris HCL (pH 8), 150 nM NaCl, 0.1% SDS, 1% Triton X-100, 1% natriumdeoxycholate, 1× protease inhibitor cocktail, 1 mM PMSF, 50 mM NaF, 2 mM EDTA, and 1 mM Na_3_VO_4_). Moreover, HIBCPP were scraped from filter inserts, centrifuged for 10 minutes at 18000 × g and thereafter lysed as described above. The whole protein content was determined by the Lowry method (DC Protein Assay, BioRad, Hercules, CA, USA) according to the manufacturer’s instruction. The protein samples were mixed with loading buffer and reducing agent (4 to 12%) (Invitrogen). Equal amounts of protein sample were subjected to electrophoresis at 200 V (MOPS running buffer, Invitrogen). The proteins were separated on Bis Tris NuPage® gels (Invitrogen) and afterwards transferred onto a nitrocellulose membrane (0.45 μm, BioRad, USA) by blotting in transfer buffer for 16 h at 20 V and 4°C (Invitrogen). For prevention of non-specific binding of the primary antibody, the membrane was incubated in Tris-buffered saline and Tween 20 (TBST) buffer with 5% milk powder for 1 h. The primary antibodies recognizing β-actin (Sigma, USA) or SIRPα (Abcam, Cambridge, UK) respectively, were incubated overnight at 4°C, and secondary antibodies (anti-mouse and anti-rabbit) were incubated for 1 h at room temperature. The blots were analyzed using Chemi-Smart (Vilbert Lourmat, Torcy, France). For deglycosylation experiments the PNGase F Kit (BioLabs, Ipswich, MA, USA) was used according to the manufacturer’s instructions, using 5 μg or 20 μg of protein depending on the protein lysate sources. Subsequently, loading buffer was added to the deglycosylated proteins and a western blot was performed as described above.

### Cytometric bead array

For cytokine/chemokine detection, supernatants were collected immediately after the TM experiments. After centrifugation of the leukocytes, the supernatants were frozen directly at −80°C for later measurement. Samples were analyzed using the Luminex xMAP suspension array technology (Luminex, Austin, TX, USA). A commercially available kit (G-CSF, GM-CSF, GRO (CXCL1,2,3), IL-1α, IL-1β, IL-1ra, IL-6, IL-8, IP-10, monocyte chemotactic protein (MCP)-1, MDC, macrophage inflammatory protein (MIP)-1α, MIP-1β, platelet-derived growth factor (PDGF)-AA, PDGF-AB/BB, regulated and normal T cell expressed and secreted (RANTES), sCD40L, transforming growth factor (TGF)α, TNFα, vascular epithelial growth factor (VEGF; MPXHCYTO-60 K, Millipore, Germany, for PMN) was used for the detection of different cytokines/chemokines secreted in PMN experiments and another predefined panel of human cytokines/chemokines for the supernatant of monocyte experiments (Eotaxin, Fractalkline, G-CSF, GRO, IL-10, IL-1ra, IL-1α, IL-1β, IL-6, IL-8, IP-10, MIP-1α, MIP-1β, TNF-α; MPXHCYTO-60 K-14 - Human, Millipore, Germany, for monocytes) was used to quantify cytokines according to the manufacturer’s instructions. Samples (25 μl) were used undiluted and were incubated overnight. Standard curves for each cytokine (in duplicate) were generated using the reference cytokine concentrations supplied with this kit. All incubation steps were performed at room temperature in the dark to protect the beads from light. Samples were read on the Luminex 100™ system (Luminex). To avoid between-run imprecision, all samples from the same individual before and after the interventions were measured in the same run. Control samples were included in all runs. The detection limit for any analyte was 3.2 pg/ml with a dynamic range up to 10,000 pg/ml (according to the manufacturer`s instruction). A variety of control samples was included in all runs. The final concentrations were calculated as the average of two independent measures using the IS software version 2.3.

### Measurement of cell viability

Cell vitality was measured using the Live/Dead® Viability/Cytotoxicity Kit for mammalian cells (Molecular Probes, Göttingen, Germany) according to the manufacturer’s instructions. The results were photo-documented by fluorescence microscopy. Cytotoxicity was not observed in any of the experiments (data not shown).

### Statistical analysis

All statistical calculations have been performed using the SAS system, release 9.2 (SAS Institute Inc., Cary, NC, USA). Quantitative parameters are presented as mean values and SD. For approximately normally distributed data (for resistance or TM rate), one-way analysis of variance (ANOVA) was performed to compare the mean values between several groups. In the case of a significant test result, pairwise comparisons were made using Scheffé’s test, or (to compare to a single control) Dunnett’s test. For these analyses, the SAS procedure PROC MIXED was used with the experiment and amount of filters used as random factors. For skewed data (on permeability), the Kruskal-Wallis test was used followed by the Mann–Whitney *U*-test for pairwise comparisons. A test result was considered statistically significant for *P*-values below 0.05.

## Results

To investigate the TM of leukocytes across the human BCSFB, an inverted transwell system was used, which has been described in previous studies with PCPEC and HIBCPP [2,16]. With this system it is possible to analyze the TM of leukocytes across or through epithelial cells from the physiologically relevant basolateral blood side to the apical CSF side.

### *N*. *meningitidis* stimulates PMN transmigration across HIBCPP

**Figure 1:**
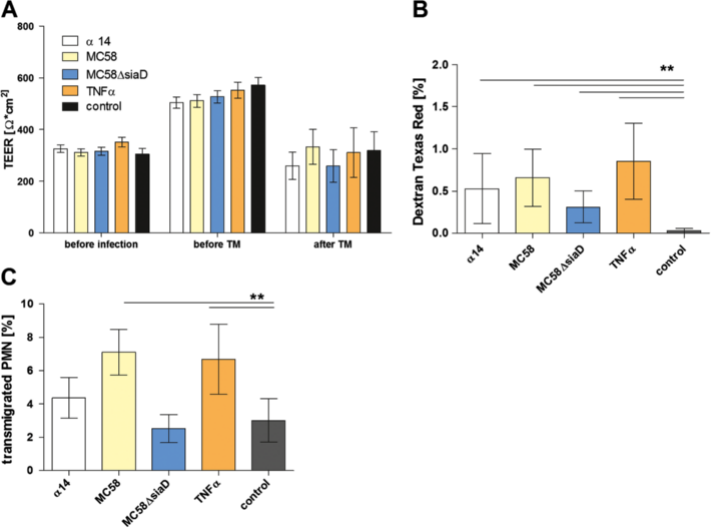
**Effects of *N.meningitidis *on polymorphonuclear neutrophil (PMN) transmigration (TM) through human choroid plexus papilloma cells (HIBCPP).** HIBCPP were infected with *N*. *meningitidis* MC58 wild-type, its unencapsulated mutant MC58ΔsiaD, or with the apathogenic strain α14 (multiplicity of infection = 10) for 2 h, or stimulated with TNFα (10 ng/ml) as described in Material and methods. PMNs were applied 24 h after stimulation onto the basolateral side of the inverted HIBCPP culture (PMN:HIBCPP ratio of 10:1). During the experiment the effect on barrier function of the HIBCPP layer was determined by transepithelial electrical resistance (TEER) (**A**) and dextran TexasRed flux (**B**). TEER values were measured before stimulation, 24 h thereafter, and 4 h after the PMN TM period (**A**) (n = 9 in triplicate). Dextran TexasRed flux was measured in the basolateral-to-apical direction after the TM period of 4 h (**B**) (n = 5 in triplicate). In this experimental setting IL-8 (10 ng/ml) was used as chemoattractant, which was applied to the basolateral side of the inverted transwell HIBCPP culture. The percentage of transmigrated PMNs was measured fluorometrically after 4 h of TM (n = 3 in triplicate). ^**^P <0.01 compared to corresponding control HIBCPP.

First we analyzed the effect of PMN on the HIBCPP barrier function with or without bacterial stimulation. We observed an overall increase of TEER after 24 h of stimulation under all conditions (Figure 1A). Thereafter, PMNs were applied in the upper compartment of the culture insert with or without additional IL-8 in the lower filter compartment. We observed a significant increase in PMN TM in the presence of IL-8 (3.01% ± 0.45%) compared to the TM without IL-8 (1.5% ± 0.37%) (data not shown).

Next we analyzed barrier function and TM of PMNs after infection of HIBCPP with *N*. *meningitidis*. After the 4-h PMN TM period we observed a significant TEER decrease under all conditions (Figure 1A). The paracellular permeability remained low under all conditions, but was significantly lower under control conditions compared to stimulated cells (Figure 1B). Importantly, we demonstrated a significant increase in TM after stimulation with strain MC58 and TNFα, whereas the TM remained in the range of control values after stimulation with strain MC58ΔsiaD and strain α14 (Figure 1C). In Live/Dead assays no significant alteration of HIBCPP or PMN viability was observed (data not shown).

### *N*. *meningitidis* inhibitis monocyte transmigration across HIBCPP

**Figure 2:**
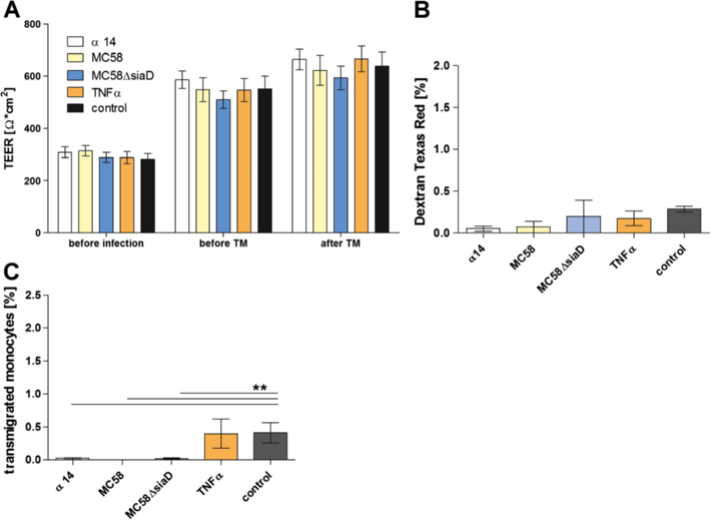
**Effects of *N.meningitidis *on monocyte transmigration through human choroid plexus papilloma cells (HIBCPP).** HIBCPP were infected with *N*. *meningitidis* MC58 wild-type, its unencapsulated mutant MC58ΔsiaD or with the apathogenic strain α14 (multiplicity of infection = 10) for 2 h, or stimulated with TNFα (10 ng/ml) as described in Materials and methods. PMNs were applied 24 h after stimulation onto the basolateral side of the inverted HIBCPP culture (monocyte:HIBCPP ratio of 1:1). During the experiment the effect on barrier function of the HIBCPP layer was determined by transepithelial electrical resistance (TEER) (**A**) and dextran TexasRed flux (**B**). TEER values were measured before stimulation, 24 h thereafter and 4 hours after the monocyte TM period (**A**) (n = 5 in triplicate). Dextran TexasRed flux was measured in the basolateral-to-apical direction after the TM period of 4 h (**B**) (n = 5 in triplicate). In this experimental setting, monocyte chemoattractant protein (MCP) -1 (50 ng/ml) was used as chemoattranctant for the 2’,7’-bis-(2-carboxyethyl)-5-(and-6)-carboxyfluorescein (BCECF)-labeled monocytes, which were applied to the basolateral side of the inverted transwell HIBCPP culture. The percentage of transmigrated monocytes was measured fluorometrically after 4 h of TM (n = 5 in triplicate). ^**^P <0.01 compared to corresponding control HIBCPP.

In the next step we analyzed monocyte transmigration across HIBCPP and its effect on the barrier function after infection with *N*. *meningitidis*. First we investigated monocyte TM in the presence or absence of the chemoattractant MCP-1 (0.046% ± 0.075%) and demonstrated a significant increase in monocyte TM in the presence of MCP-1 (1.02% ± 0.031%) (data not shown). After bacterial infection of HIBCPP and monocyte TM we observed no significant change in barrier function (Figure 2A, B). In contrast to the PMN TM, the migration of monocytes was significantly inhibited by bacterial infection, whereas TNFα had no influence compared to unstimulated controls (Figure 2C). No significant alteration of HIBCPP or monocyte viability was observed (data not shown).

### Increased monocyte transmigration after bacterial stimulation in the presence of PMNs

**Figure 3:**
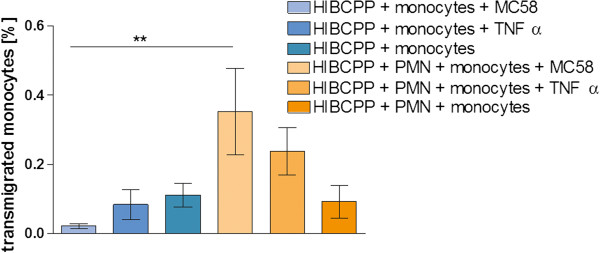
**Sequential transmigration (TM) of polymorphonuclear neutrophils (PMNs) and monocytes through human choroid plexus papilloma cells (HIBCPP).** Shown is a sequential TM experiment comparing the TM of monocytes alone through HIBCPP with a co-culture of PMNs with monocytes. PMNs were added to the basolateral cell side 24 h after bacterial infection, followed by the application of 2’,7’-bis-(2-carboxyethyl)-5-(and-6)-carboxyfluorescein, acetomethyl ester (BCECF-AM)-labeled monocytes 2 h later. Monocyte TM alone was analyzed in parallel without PMNs for 2 h (n = 3 in triplicate). ^**^P <0.01 compared to stimulated monocyte TM without PMN.

During bacterial meningitis PMNs display the first line of immune defense followed by monocyte infiltration as the second wave of inflammatory response [35]. Consequently, we studied whether the presence of PMNs can influence the TM of monocytes over the plexus epithelium after bacterial infection with *N*. *meningitidis*. Additionally, as known from previous work, PMN granule proteins can promote monocyte recruitment and work as chemoattractants inducing also the secretion of MCP-1 or IL-8 [36]. Therefore we performed co-culture PMN/monocyte TM experiments. PMNs were applied for 2 h into the apical transwell filter compartment; afterwards monocytes were added for another 2 h and the final TM rate of monocytes was analyzed. In these co-culture experiments monocyte TM after bacterial stimulation with MC58 was significantly increased in the presence of PMN compared to monocyte TM alone (Figure 3).

### PMNs and monocytes migrate via the para- and transcellular route through HIBCPP

**Figure 4:**
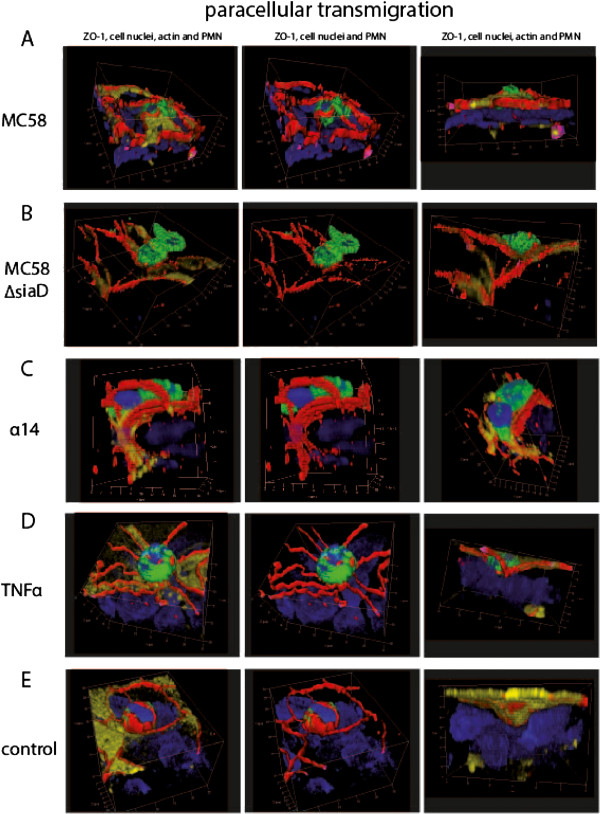
**Paracellular polymorphonuclear neutrophils (PMN) traversal across human choroid plexus papilloma cells (HIBCPP).** HIBCPPs were stained for the tight junction (TJ) protein zonula occludens (ZO)-1 (red), actin (phalloidin, yellow), cell nuclei (4‘-6-diamidino-2-phenylindole dihydrochloride (DAPI), blue), and additionally we stained for the PMN marker (CD66b-fluorescein isothiocyanate (FITC), green). (**A**-**E**) Three-dimensional immunofluorescence images of HIBCPP and transmigrating PMNs reconstructed from 0.3 μm Apotome® optical sections, using Zeiss software Inside 4D. The views from above and the side views show paracellular transmigration, where PMNs are migrating through the intercellular spaces. Images show the transmigration of PMNs through HIBCPP after previous stimulation with strain MC58 (**A**), strain MC58ΔsiaD (**B**), strain α14 (**C**), TNFα (**D**), and control conditions (**E**). The figures show representative examples of three independent experiments that all gave similar results. Scale bar as indicated.

**Figure 5:**
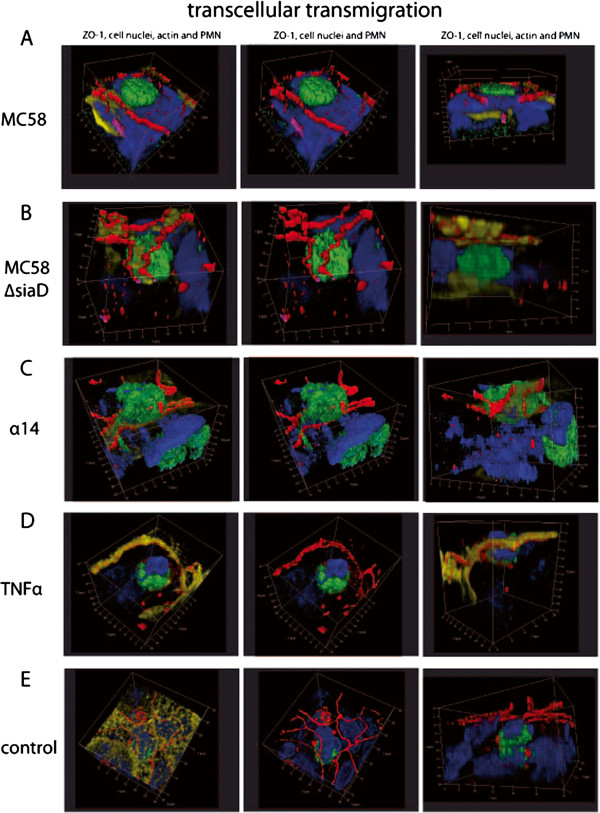
**Transcellular polymorphonuclear neutrophils (PMN) traversal across human choroid plexus papilloma cells (HIBCPP).** HIBCPPs were stained for the tight junction (TJ) protein zonula occludens (ZO)-1 (red), actin (phalloidin, yellow), cell nuclei (4‘-6-diamidino-2-phenylindole dihydrochloride (DAPI), blue), and in addition we stained PMNs for a neutrophil marker (CD66b- fluorescein isothiocyanate (FITC), green). (**A**-**E**) Three-dimensional immunofluorescence images of HIBCPP and transmigrating PMNs reconstructed from 0.3 μm Apotome® optical sections, using Zeiss software Inside 4D. The views from above and the side views show a transcellular non-junctional transmigration pathway where the PMNs migrate in a clear distance to the cell borders. Images show the transmigration of PMNs through HIBCPP after previous stimulation with strain MC58 (**A**), strain MC58ΔsiaD (**B**), strain α14 (**C**), TNFα (**D**), and control conditions (**E**). The figures show representative examples of three independent experiments that all gave similar results. Scale bar as indicated.

In the following experiments we analyzed the TM pathway used by the PMNs and monocytes to overcome the plexus epithelium. Therefore, we performed extensive immunofluorescence, and electron microscopic studies, as well as FIB/SEM studies to elucidate the TM process in more detail. Three-dimensional immunofluorescence analysis revealed that PMNs migrate via the para- (Figure 4) and transcellular (Figure 5) route through HIBCPP. The first immunofluorescence series shows paracellular migrating PMNs, which squeeze through the TJs (Figure 4A-E). In Figure 4A the PMN is about to exit the intercellular space, where the ZO-1 strands are interrupted; in Figure 4B and C the PMN has almost left the HIBCPP cell through a widened intercellular space. In the latter image the intercellular gap formation was observed almost from the top to the lower level of the HIBCPP cell. In Figure 4D the PMN is squeezing between TJs at a multicellular corner and in Figure 4E the PMN is covered by ZO-1 from two sides in parallel, indicating direct traversal through the tight junctions.

In the case of transcellular TM, PMNs migrated in a clear distance to the cell borders (Figure 5). Here, no TJ alterations were found in different stages of PMN traversal through the epithelial cell from the basolateral to the apical side. As seen in Figure 5A and D, the migrating PMNs are found above the cell level with only a part within the cell. The side views illustrate that the PMN is about to leave the cell. In Figure 5C the major part of the PMN is found already outside of the cell and the PMN is surrounded by TJ strands, whereas in Figure 5B and E the PMN is still located within the HIBCPP cell body.

**Figure 6:**
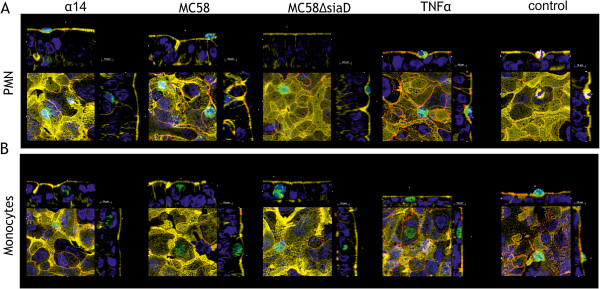
**Actin cytoskeleton morphology in HIBCPP after immune cell transmigration.***En face* Apotome® microscopy images, showing human choroid plexus papilloma cells (HIBCPPs) stained for the tight junction (TJ) protein zonula occludens (ZO)-1 (red), the actin (phalloidin, yellow) and cell nuclei (4‘-6-diamidino-2-phenylindole dihydrochloride (DAPI), blue), polymorphonuclear neutrophils (PMNs) stained with a neutrophil marker (CD66b-fluorescein isothiocyanate (FITC), green) and monocytes labeled with CD16-FITC (green). Shown is actin cytoskeleton morphology of HIBCPP after PMN (**A**) and monocyte (**B**) transmigration under different simulation conditions (α14, MC58, MC58ΔsiaD, TNFα and control conditions). Actin cytoskeleton analysis showed no major differences in actin morphology between control and stimulated HIBCPP cells as well as in PMN or monocyte experiments. Of note, the visualized monocytes are obsverved very rarely especially after bacterial stimulation. The figures show representative examples of three independent experiments that all gave similar results. Scale bar as indicated.

In the next step the actin morphology during this TM process of PMN and monocytes was further analyzed, showing no differences between stimulated HIBCPP cells and control conditions (Figure 6). Moreover no major differences of actin morphology in PMN and monocyte experiments were observed. Interestingly, HIBCPP showed a tubular morphology after bacterial stimulation, in contrast to TNFα-stimulated and control cells, which had a rather flat character.

**Figure 7:**
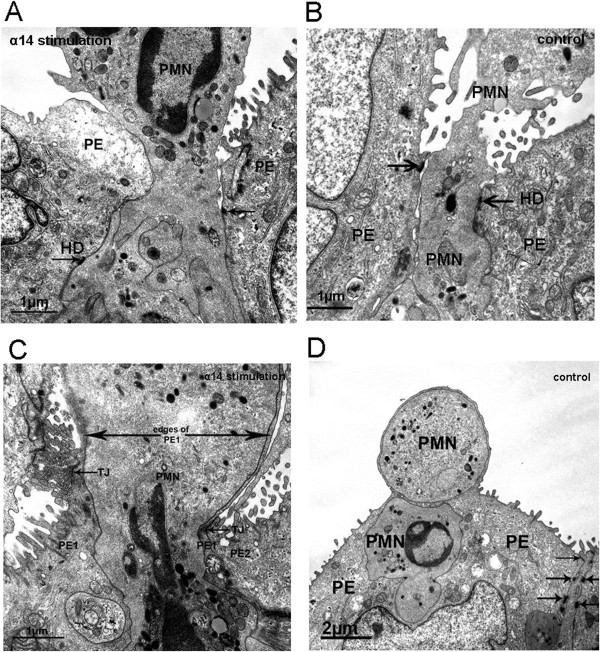
**Transmission electron microscopic analysis of polymorphonuclear neutrophils (PMNs) migrating through human choroid plexus papilloma cells (HIBCPP).** (**A**, **B**) Cross-sections of paracellular-migrating PMNs. The arrows indicate the hemidesmosomes. (**C**, **D**) Transcellular migrating PMNs through strain α14-stimulated and control HIBCPP. PMNs are migrating in a clear distance to the cell borders; the intact tight junctions (TJs) are marked with arrows. (**C**) The PMN migrates through the plexus epithelial cell (PE1) and is separated by a small cell bridge to another cell (PE2). Scale bar as indicated. These figures are representative for all other conditions.

Electron microscope investigations were performed to provide further evidence for the mode of TM of PMN through the HIBCPP monolayer. We compared ortho-graded filters that were sectioned almost parallel through the apical surface, to allow better interpretation of cellular details during TM. Here, as in the immunofluorescence analyses, we showed that PMNs can migrate either via the paracellular route (Figure 7A, B) or via the transcellular route through the epithelial cell itself (Figure 7C, D). In Figure 7A and B the PMN is squeezing through the paracellular space of two epithelial cells indicated by open hemidesmosomes, which are marked with arrows on each side. Figure 7C and D show intact TJs, which are marked with an arrow underlying the clear distance to the PMN.

**Figure 8:**
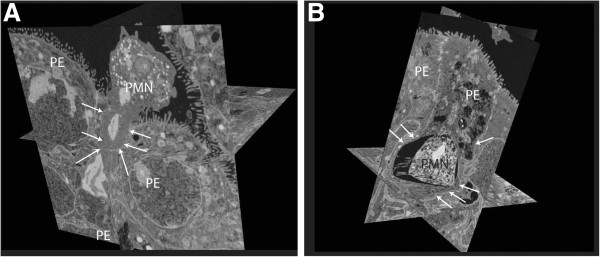
**Focused ion beam-scanning electron microscopy (FIB-SEM) CrossBeam analysis of polymorphonuclear neutrophils (PMNs) migrating through human choroid plexus papilloma cells (HIBCPP).** FIB-SEM CrossBeam analysis demonstrates (**A**) paracellular- and (**B**) transcellular-migrating PMN through HIBCPP. (**A**) Multiple crosssections (orthoslices) through the three-dimensional images. The PMN is migrating paracellular through a widened intercellular space (marked with arrows) and squeezing between two cells. (**B**) The transcellular migrating PMN is distant to the intact tight junctions (TJs) and is surrounded by the cytoplasm of the HIBCPP cell. PE, plexus epithelial cell.

With the help of the FIB/SEM Crossbeam analysis, paracellular-migrating PMN (Figure 8A Additional file 1: video 1) and transcellular-migrating PMN (Figure 8B Additional file 2: video 2) were also identified. This technique provides the possibility of three-dimensional electron microscopic analysis of the whole cell monolayer. Here we show a PMN, which migrates via the paracellular route between the epithelial plexus cells (Figure 8A) through the TJs (arrows indicate the cell borders), as well as in a clear distance to the cell borders (arrows) directly in the cytoplasm (Figure 8B).

**Figure 9:**
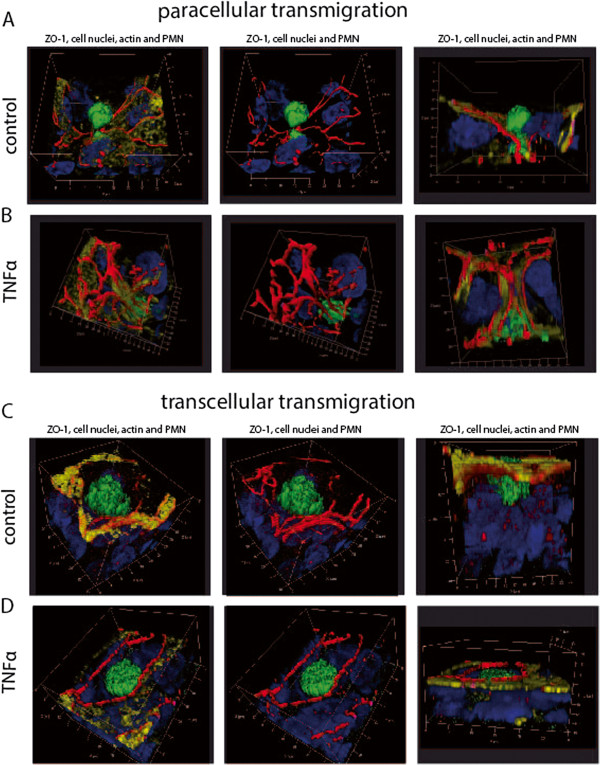
**Transcellular and paracellular monocyte traversal across human choroid plexus papilloma cells HIBCPP.** HIBCPP were stained for zonula occludens (ZO)-1 (red), actin (yellow) and cell nuclei (blue); additionally, monocytes were stained for a monocyte marker (CD14- fluorescein isothiocyanate (FITC), green). (**A**-**D**) Three-dimensional immunofluorescence images of HIBCPP and transmigrating monocytes reconstructed from 0.3 μm Apotome optical sections, using Zeiss software Inside 4D. The images show different transmigration routes. Para- and transcelluarly migrating monocytes are shown after TNFα stimulation (**A**, **C**) and under control conditions (**B**, **D**). The figure shows a respresentative example of three independent experiments that all gave similar results. Scale bar as indicated.

Due to the low TM rate of monocytes, which was significantly reduced after bacterial infection, the usefulness of immunofluorescence and especially, electron microscopic analysis to clarify the TM route of monocytes was limited. Therefore, we focused on immunofluorescence studies of TNFα-stimulated and unstimulated control HIBCPP (Figure 9). In control and TNFα-stimulated cells we observed paracellular-migrating monocytes, which were frequently found to migrate at bi- or multicellular corners (Figure 9A, B) as well as transcellular ones (Figure 9C, D). In Figure 9A the monocyte is exiting the cell via the paracellular route, whereas Figure 9B displays a monocyte entering the paracellular migration route. Furthermore transcellular transmigrating monocytes were found in the HIBCPP cell body and at a clear distance from the cell borders (Figure 9C, D).

### Inflammatory response after leukocyte transmigration across HIBCPP

**Figure 10:**
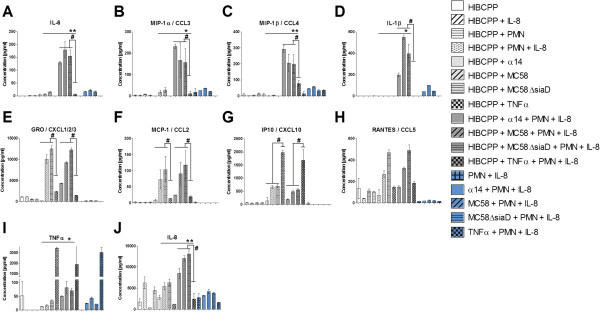
**Cytokines and chemokines released during polymorphonuclear neutrophils (PMN) transmigration (TM).** The supernatants of transwell filters were analyzed after PMN TM experiments for different cytokines/chemokines (pg/ml) with cytokine bead array (CBA) after stimulation of human choroid plexus papilloma cells (HIBCPP) with strain α14, strain MC58, strain MC58ΔsiaD or TNFα. The cyotokines IL-6, macrophage inflammatory protein (MIP)-1α, MIP-1β, IL-1β were strongly secreted after bacterial infection of HIBCPP and PMN TM (**A**-**D**), whereas GRO (CXCL1,2,3), monocyte chemoattractant protein (MCP)-1, Interferon gamma-induced protein-10 (IP-10) and RANTES (regulated and normal T cell expressed and secreted) were already secreted after bacterial stimulation of HIBCPP alone (**E**-**H**). TNFα and IL-8 were also released during PMN transmigration and after infection of HIBCPP (**I**-**J**). Data shown are means ± SD of two independent experiments and two filters per condition analyzed in duplicate. ^*^P <0.05, ^**^P <0.01 compared to stimulated HIBCPP with and without PMNs. ^#^*P* <0.05 compared to TNFα-stimulated cells.

To analyze whether a distinct cytokine/chemokine pattern is responsible for the differential TM pattern of PMNs and monocytes a cytometric bead array was performed with supernatants of PMN and monocyte experiments as described above. The first group of cytokines/chemokines including IL-6, MIP-1α, MIP-1β and IL-1β showed significantly increased secretion levels after the TM of PMNs through infected HIBCPP (Figure 10A-D), whereas another group of cytokines such as GRO, MCP-1, IP-10 and RANTES (Figure 10E-H) was shown to be released by HIBCPP already after stimulation with different *N*. *meningitidis* stains alone. TNFα was used again as the positive inflammatory control. TNFα (Figure 10I) and IL-8 (Figure 10J) were significantly produced after bacterial stimulation of HIBCPP, which was further enhanced after the PMN TM.

**Figure 11:**
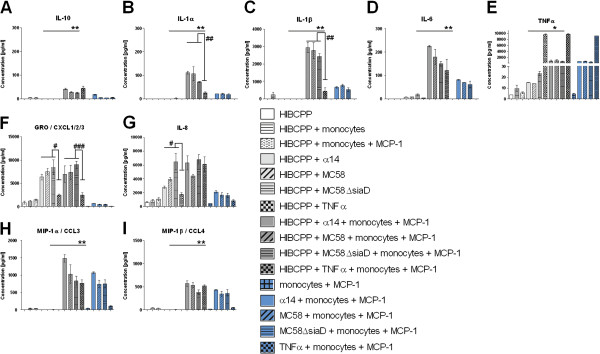
**Cytokines and chemokines released during monocyte transmigration.** The supernatants of transwell filters were analyzed after monocyte TM experiments for different cytokines/chemokines (pg/ml) with cytokine bead array (CBA) after stimulation of human choroid plexus papilloma cells (HIBCPP) with strain α14, strain MC58, strain MC58ΔsiaD or TNFα. IL10, IL1α, IL1β, IL-6 and TNFα display a group of cytokines mainly secreted after monocytes have migrated through infected HIBCPPs (**A**-**E**). GRO (CXCL1,2,3,) and IL-8 were already secreted by HIBCPP when stimulated with bacteria alone (**F**-**G**). Macrophage inflammatory protein (MIP)-1α and MIP-1β were released after bacterial stimulation of monocytes (**H**-**I**). Data shown are mean ± SD of two independent experiments and two filters per condition analyzed in duplicate. ^*^P <0.05; ^**^P <0.01; ^***^P <0.001 compared to stimulated HIBCPP with and without monocytes. ^#^*P* <0.05; ^##^*P* <0.01; ^###^*P* <0.001 compared to TNFα-stimulated cells.

As in the PMN analyses the detected cytokines/chemokines in monocyte TM experiments were classified in different categories of their secretion pattern after the different stimuli. IL10, IL1α, IL1β, IL-6 and TNFα (Figure 11A-E) were mainly released after monocyte TM in combination with bacterial stimulation, whereas GRO and IL-8 (Figure 11F-G) were already secreted at higher levels only after bacterial stimulation of HIBCPP alone. Another group displayed chemokines, such as, MIP-1α and MIP-1β (Figure 11H-I), which were strongly secreted by monocytes alone after bacterial stimulation. This secretion was further enhanced by the presence of HIBCPP.

Comparing the cytokine bead array (CBA) results of the monocyte and PMN TM experiments, we observed, that the cytokine/chemokine levels secreted during PMN migration differed significantly from those observed during monocyte migration. Chemokines, such as, MIP-1α and MIP-1β (Figure 10B,C and Figure 11H, I), as well as cytokines, such as IL-1β (Figure 10D; Figure 11C), are secreted in concentrations that were up to 10 times higher during monocyte TM in combination with bacterial stimulation, than during PMN TM. Moreover, MIP-1α, MIP-1β (Figure 10B,C and Figure 11H, I) and IL-1β (Figure 10D and Figure 11C) were also released at levels around 10 times higher after bacterial stimulation of monocytes without HIBCPP compared to PMN. Both GRO and IL-8 had similar secretion levels in both settings (Figure 10E, J and Figure 11F, G).

### SIRPα is declosylated during TM of monocytes through HIBCPP

**Figure 12:**
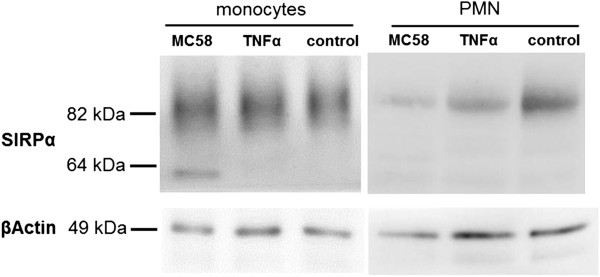
**Signal regulatory protein (SIRP)α expression on transmigrated monocytes and polymorphonuclear neutrophils (PMNs).** Lysates of monocytes and PMNs from the transmigration experiment involving stimulated (MC58, TNFα or control) human choroid plexus papilloma cells (HIBCPP) were used for western blotting. SIRPα protein levels of the neuronal (80 to 85 kDa) and its deglycosylated form (50 to 60 kDa) were determined. The figure shows a representative example of five independent experiments that all gave similar results.

In addition to the cytokine/chemokine analysis we wanted to determine possible binding partners of PMNs and monocytes involved in the TM process. As already known from previous studies, SIRPα was shown to mediate migration of leukocytes through endothelial [37,38] and epithelial cells [39]. Therefore we aimed to investigate the possible involvement of SIRPα in the differential TM of PMNs and monocytes by western blot analysis. We determined the deglycosylation of SIRPα after bacterial infection, after TNFα stimulation, and under control conditions. As shown in Figure 12 the neuronal form of SIRPα is expressed at about 85 kDa under all conditions. Interestingly, SIRPα was deglycosylated in monocytes after bacterial infection, whereas under TNFα stimulation and under control conditions only a very slight band of the deglycosylated form was observed. In contrast, only the neuronal form of SIRPα was observed in PMN experiments, as well as in experiments with HIBCPP only (data not shown).

## Discussion

The plexus epithelium displays a potential entry site for leukocytes during bacterial meningitis [8]. In this study we investigated for the first time the cellular and molecular mechanism of the TM process of PMNs and monocytes through the human BCSFB after bacterial infection *in vitro*. We could demonstrate an increased PMN TM rate after infection with *N*. *meningitidis* strain MC58, whereas the same stimulus inhibited monocyte TM. In contrast, in co-culture experiments with PMNs, monocytes were facilitated to migrate in significantly higher levels. Moreover, we showed that PMNs as well as monocytes can migrate via the transcellular or paracellular route through HIBCPP. Finally, our data provide evidence that a distinct cytokine and chemokine pattern as well as SIRPα may be involved in the PMN and monocyte TM process, respectively.

Recently, an HIBCPP was established [24] and further characterized as a BCSFB *in vitro* model [10]. PMN migration into the CSF as the first wave of immune defense displays an important part of the pathogenesis of bacterial meningitis, leading to inflammation, followed by the recruitment of monocytes [40]. Our model mimics the *in vivo* situation, by applying bacteria and leukocytes to the basolateral side of the host cell and allows analysis of the TM in the *in vivo* relevant direction from the basolateral to the apical cell side [2].

Using HIBCPP as a model system for the BCSFB we observed robust barrier properties, even though the TEER was slightly influenced by PMN TM, and the paracellular permeability by bacterial and TNFα stimulation. However, a change of TEER does not necessarily correlate with paracellular flux and also depends on the size of TJ tracer used [41]. As we have used dextran in our study, this molecule is not as sensitive as mannitol, for example. Moreover, a change of TEER or paracellular dextran flux does not necessarily correlate with leukocyte TM [41]. The specific stimulus can either modify TEER, paracellular flux, or both. In our particular case, where PMN and monocytes also migrated to a large extent via the transcellular route, an alteration of TJ function was not necessarily expected. Additionally, the increased dextran flux alone might not lead to paracellular TM of PMNs, since the observed compromise of TJ function was still much lower than that of our own previously published results in the standard transwell system after *Streptococcus suis* (*S*. *suis*) infection [3,42]. Conflicting data exist as to whether PMN TM itself can lead to significant barrier disruption, and this seems to depend on the cells and the stimulus used [41,43-45].

In PCPEC and human brain microvascular endothelial cells (HBMEC) [16,43] it has already been shown that PMN TM can cause a barrier alteration. The PMN TM rate was significantly increased after infection with the capsule expressing strain MC58 and TNFα stimulation compared to infection with the unencapsulated mutant strain MC58ΔsiaD, strain α14 and control conditions. TNFα has already been known to influence leukocyte cell migration over renal tubular, intestinal epithelial and choroid plexus epithelial cells [16,46,47]. The role of different *Neisseria meningitidis* strains in leukocyte TM has not been investigated in previous studies. Recently, we demonstrated in a porcine BCSFB model that infection of PCPEC with the encapsulated wild-type strain 10 of *S*. *suis* led to an increased TM rate of porcine PMNs [16]. Of note, the entry of *S*. *suis* and *N*. *meningitidis* into HIBCPP is attenuated by the presence of the capsule [10]. Therefore, the presence of the capsule may induce cell signaling in HIBCPP, which may promote an increased TM rate and vice versa.

In alveolar epithelial cells infection of *Moraxella catarrhalis* was shown to induce monocyte adhesion, transepithelial migration and superoxidegeneration, whereas stimulation with lipopolysaccharide (LPS), TNFα, IL-1β or IFN-γ induced adhesion or TM. Therefore, we assumed that an increased proinflammatory response after infection with *N*. *meningititis* would also promote monocyte TM across HIBCPP [48]. However, neither the infection with the encapsulated wild-type strain MC58 nor the unencapsulated MC58ΔsiaD or strain α14 induced TM of monocytes across HIBCPP layers. Therefore, the bacterial stimulation itself, rather than the capsule had an influence on the reduced TM rate. In contrast, the stimulation with the proinflammatory cytokine TNFα and control conditions led to a significant higher monocyte TM compared to bacterial stimulation. Interestingly, it has previously been shown that the encapsulated meningococci induce lower levels of proinflammatory cytokines, such as TNFα and IL-6, in THP-1 monocytes [49]. In our study, encapsulated as well as unencapsulated strains caused a similar inflammatory response in monocytes.

From clinical observations it is known from sequential lumbar punctures that PMNs display the first line of immune defense in the CSF. But within 24 h after PMN influx mononuclear cells increase in number representing the second wave of inflammation [35,40]. In studies with mice it has been demonstrated that PMNs interacting with the endothelium release soluble factors, such as, azuorcidin, LL-37, and cathepsin G, which initiate the monocyte recruitment to the side of inflammation, demonstrating the dependence on neutrophils [50,51]. Additionally, PMN granule proteins have been shown to promote *de novo* synthesis of monocyte-attracting chemokines such as MCP-1 and MIP-1α by neighboring endothelial cells and macrophages. In the presence of appropriate stimuli (for example, fMLP, TNFα, LPS), PMNs can produce and secrete monocyte-attracting chemokines themselves [51]. In the human BCSFB the sequential TM of leukocytes has not been studied *in vitro* or *in vivo*. To analyze this phenomenon for epithelial cells, we performed a sequential PMN-monocyte TM experiment with HIBCPP. Our results showed PMNs promoting the monocyte TM even after bacterial infection. In future experiments the mechanisms for these observations have to be identified. Also in recent analysis of rat choroid plexus tissue evidence was provided for the movement of monocytes after traumatic injury, sometimes in tandem with neutrophils, along the paracellular pathways between adjacent epithelial cells [52].

For transendothelial migration, a paracellular route between adjacent cells has been postulated for a long time, but in the meanwhile the transcellular route directly through the endothelial cell body has been well documented [15]. Until lately, there was no evidence that PMNs take the transcellular route through human epithelial cells; only paracellular-migrating PMNs have been observed over intestinal and lung epithelial cells [53]. However, electron microscopic analysis in PCPEC revealed that paracellular migration of PMNs stopped just in front of TJs. Interestingly, PMNs subsequently appeared to proceed by transcellular migration via funnel-like structures developing from the apical membrane [16].

In the current study we provide evidence for paracellular as well as for transcelluar TM of PMNs and monocytes through the human BCSFB with the help of three-dimensional immunofluorescence imaging, transmission electron microscopy and the FIB-SEM technique. We found PMNs and monocytes migrating paracellularly between the cells, which were surrounded by TJ proteins and actin bundles, and observed TJ alterations at the site of migration. Transcellular-migrating PMNs or monocytes were found at a clear distance from the cell borders, migrating directly through the cytoplasm, inducing no TJ alteration. Actin cytoskeleton analysis showed no major differences in actin morphology between control and stimulated HIBCPP cells, as was the case in PMN or monocyte experiments.

A thorough insight into the TM process itself was given by the transmission electron microscopy (TEM) analysis and FIB-SEM technique showing three-dimensional images with transmission electron microscope resolution displaying transcellular- and paracellular-migrating PMNs. Recently this new technique has been shown to display small features, so far only visible in TEM, which can be resolved with the FIB/SEM in a three-dimensional image with the same resolution [30]. The concert of experimental techniques applied in this study consistently suggested no preference for neutrophils or monocytes for transcellular TM pathways across HIBCPP, which is in contrast to the results in the porcine system. In PCPEC we could demonstrate a clear preference for the transcellular transepithelial migration [16].

Next we analyzed the molecular mechanisms of the leukocyte TM process. Cytokines and chemokines, including IL-8, IL-6, TNFα, MCP-1 and IL-1β, have been found in CSF during bacterial meningitis [54,55]. The CP itself has been shown to be involved in the production of cytokines/chemokines during autoimmune encephalitis as well as after infection of PCPEC with *S*. *suis*[56,57]. Protective upregulation of IL-1 and IL-10 during CNS inflammation caused by *Toxoplasma gondii* has previously been demonstrated in mice [58]. We hypothesized that a distinct cytokine/chemokine pattern may be responsible for the differential PMN or monocyte TM rate. After PMN TM we observed significantly higher secretion levels of IL-6, MIP-1α, MIP-1β, IL-1β, GRO, MCP-1 and IL-8 after bacterial stimulation compared to TNFα stimulation. Also, with monocyte TM, IL-1α, IL-1β and GRO were secreted in significantly higher levels after bacterial compared to TNFα stimulation.

Interestingly, IL-1β was mainly secreted after bacterial infection of HIBCPP and PMN TM, but not after stimulation of PMNs alone. IL-1β is known to be an important proinflammatory cytokine, which activates the immune defence [59]. Also, IL-1β promotes the inflammatory response by the release of proteins such as TNFα, which we have used as a positive control in our experiments. In monocyte TM experiments bacterial stimulation during TM induced 10-fold higher IL-1β levels than PMN experiments. Moreover, IL-1β was significantly released already after bacterial stimulation of monocytes alone. Of note, significantly less IL-1β was released after stimulation with TNFα after PMN or monocyte TM. Previously, in different models with THP-1 cells [60] and mouse macrophages [61], LPS, an important virulence factor of *N*. *meningitidis*, led to a significant release of IL-1β [59]. In the different experiments MIP-1α and MIP-1β, as well as TNFα, were also released at higher levels in the presence of monocytes but not PMNs. However, other cytokines, such as IL-6 and IL-8, showed a similar secretion pattern during PMN and monocyte experiments. Further experiments have to clarify the role of cytokines/chemokines and especially of IL-1β in the TM process [47].

Since the observed differential cytokine/chemokine response in our experiments could not unequivocally explain the reduced TM rate of monocytes after bacterial stimulation, we analyzed SIRPα as a potential binding partner of monocytes to the epithelial cells. SIRPα is of importance in the initial adhesion step of leukocyte migration over epithelial [62] or endothelial cells [37,63]. Previous studies have revealed that the degree of glycosylation of SIRPα influences the ability to bind CD47 [21,22]. In monocyte experiments presented here we demonstrated higher levels of deglycosylated SIRPα in monocytes after bacterial stimulation with MC58 wild-type. In comparison, hardly any deglycosylated SIRPα could be observed in PMNs and HIBCPP. Therefore, we propose a possible role of the glycosylation state for the regulation of monocyte TM over the BCSFB in our *in vitro* model. However, this mechanism may rather apply for paracellular TM, because CD47 is located at the basolateral cell side. Since we observed para- as well as transcellular-migrating monocytes mainly after TNFα stimulation and under control conditions, it remains speculative whether monocytes prefer the paracellular route after bacterial stimulation

## Conclusion

This study demonstrates a differential TM of PMNs and monocytes after infection with *N*. *meningitidis* in a human BCSFB *in vitro* model. Furthermore, we demonstrated for the first time in a human system that PMNs as well as monocytes can migrate para- and transcellularly through human epithelial cells. Moreover, we were able to display mechanisms of PMN and monocyte TM through the BCSFB, which could be relevant for therapeutic strategies in controlling CNS inflammation.

## Abbreviations

ANOVA: Analysis of variance; BBB: Blood–brain barrier; BCECF-AM: 2’,7’-bis-(2-carboxyethyl)-5-(and-6)-carboxyfluorescein acetomethyl ester; BCSFB: Blood-cerebrospinal fluid barrier; BSA: Bovine serum albumin; CBA: Cytokine bead array; CNS: Central nervous system; CP: Choroid plexus; CSF: Cerebrospinal fluid; DAPI: 4‘-6-diamidino-2-phenylindole dihydrochloride; DMEM: Dulbecco’s modified Eagle’s medium; EDTA: Ethylenediaminetetraacetic acid; EsB: Energy-selected backscattered; FCS: Fetal calf serum; FIB-SEM: Focused ion beam-scanning electron microscopy; FITC: Fluorescein isothiocyanate; HBMEC: Human brain microvascular endothelial cells; HBSS: Hank's Balanced Salt Solution; HIBCPP: Human choroid plexus papilloma cells; IFN: Interferon; Ig: Immunoglobulin; IL: Interleukin; LPS: Lipopolysaccharide; MCP-1: Monocyte chemotactic protein-1; MOI: Multiplicity of infection; OD: Optical density; PBS: Phosphate-buffered saline; PCPEC: Primary porcine choroid plexus epithelial cells; PDGF: Platelet-derived growth factor; PMN: Polymorphonuclear neutrophil; RANTES: Regulated and normal t cell expressed and secreted; RIPA: Radioimmunoprecipitation assay; SIRP: Signal regulatory protein; TBST: Tris-buffered saline and Tween 20; TEER: Transepithelial electrical resistance; TEM: Transmission electron microscopy; TGF: Transforming growth factor; TJ: Tight junction; TM: Transmigration; TNF: Tumor necrosis factor; VEGF: Vascular epithelial growth factor; ZO-1: Zonula occludens

## Competing interests

The authors declare that they have no competing interests.

## Authors’ contributions

TT and US conceived and coordinated the study, and drafted the manuscript. US performed cell culture and immunofluorescence experiments. JB and HI contributed to establish appropriate cell culture conditions. HW and BS performed the electron microscopic studies. PF performed the bead arrays and CW the statistics. CS and HS both conceived the study and have been involved in drafting the manuscript. All authors have read and approved the final version of this manuscript.

## Supplementary Material

Additional file 1: Video 1 Focused ion beam-scanning electron microscopy (FIB-SEM) CrossBeam sections of paracellular polymorphonuclear neutrophil (PMN) migration through human choroid plexus papilloma cells (HIBCPP). The video shows a paracellular migrating PMN presented in Figure 7A in orthoslices. The paracellular migrating PMN is squeezing between two cells. Click here for file

Additional 2: file Video 2 Focused ion beam-scanning electron microscopy (FIB-SEM) CrossBeam sections of transcellular polymorphonuclear neutrophil (PMN) migration through human choroid plexus papilloma cells (HIBCPP). The video shows a transcellular migrating PMN presented in Figure 7B in orthoslices. The transcellular migrating PMN is migrating in a clear distance to cell borders. Click here for file
